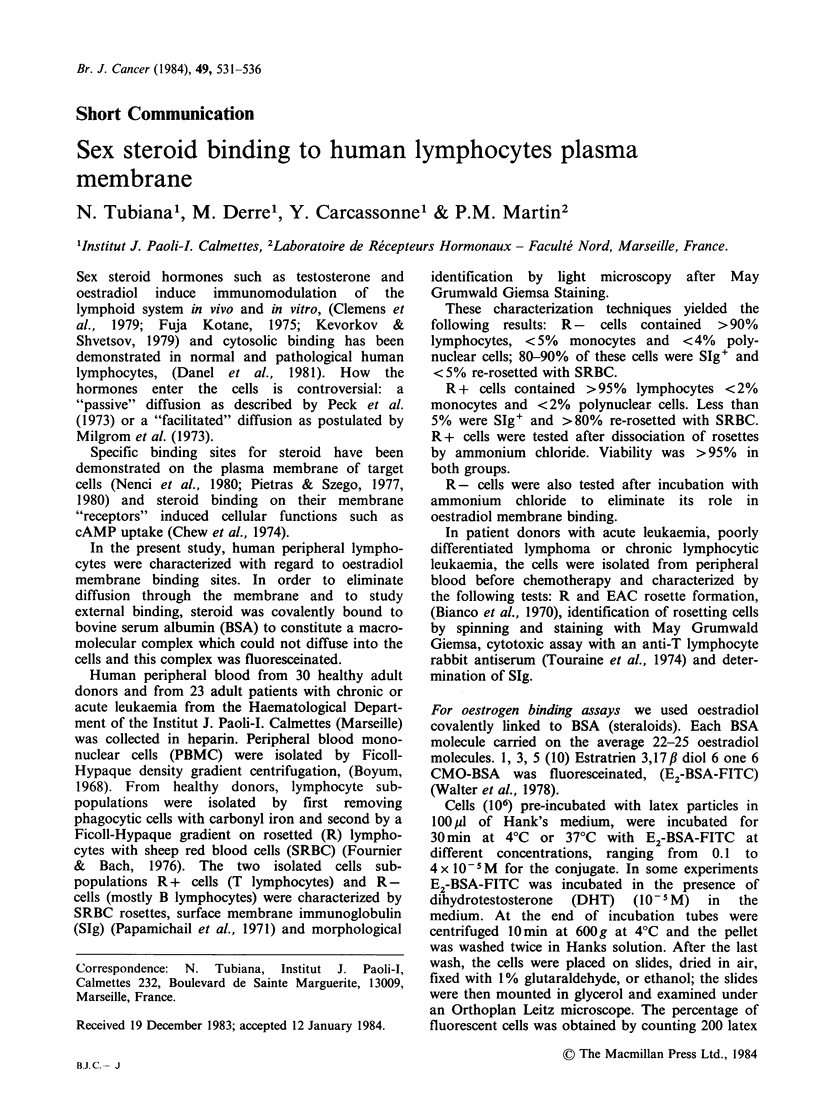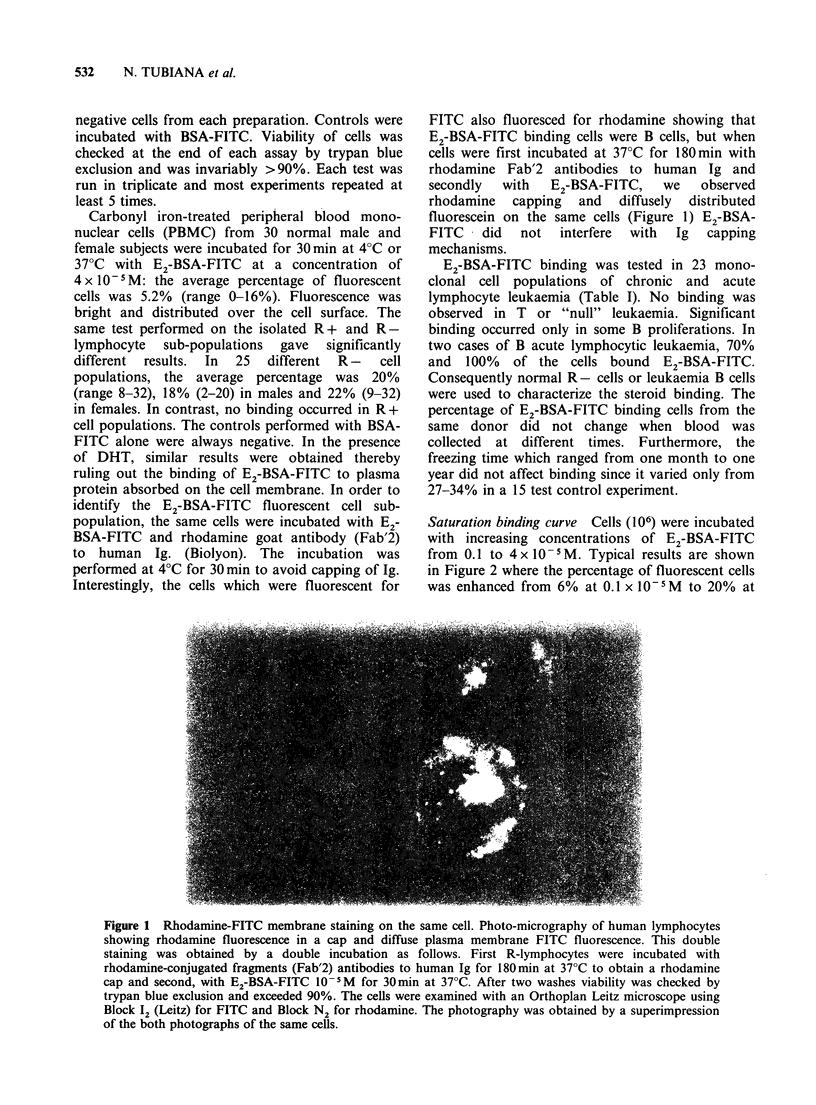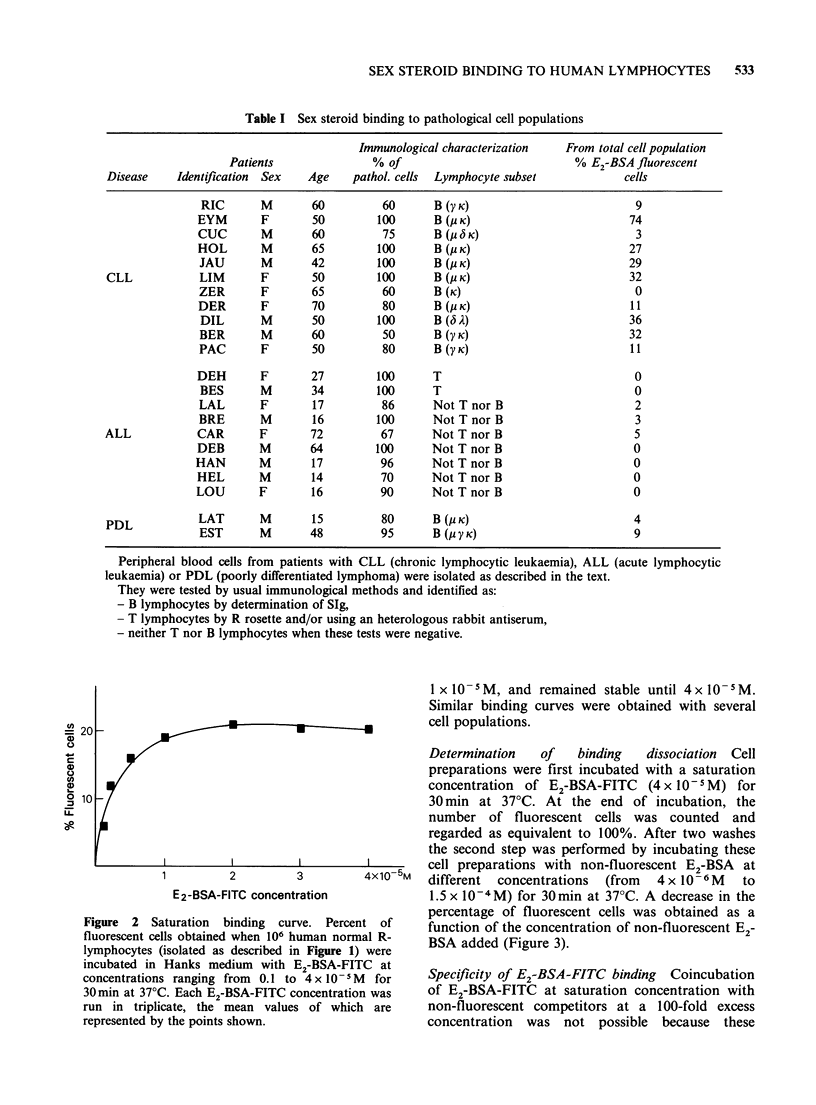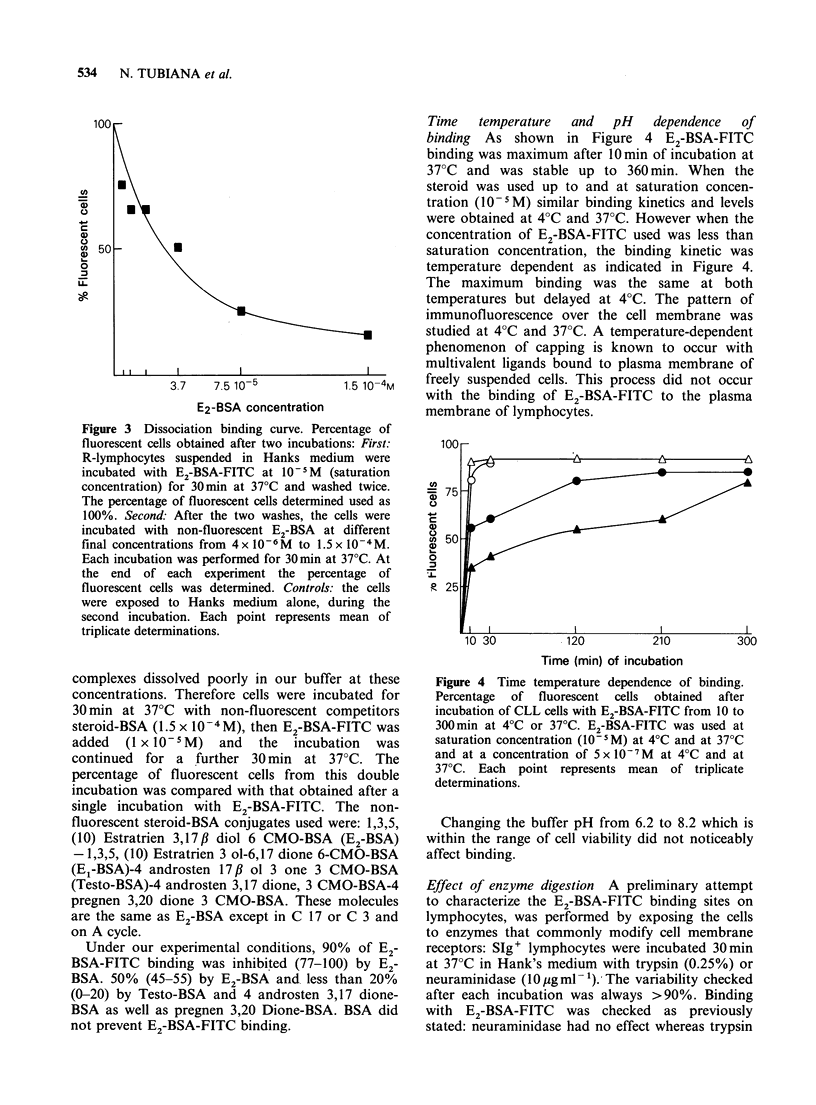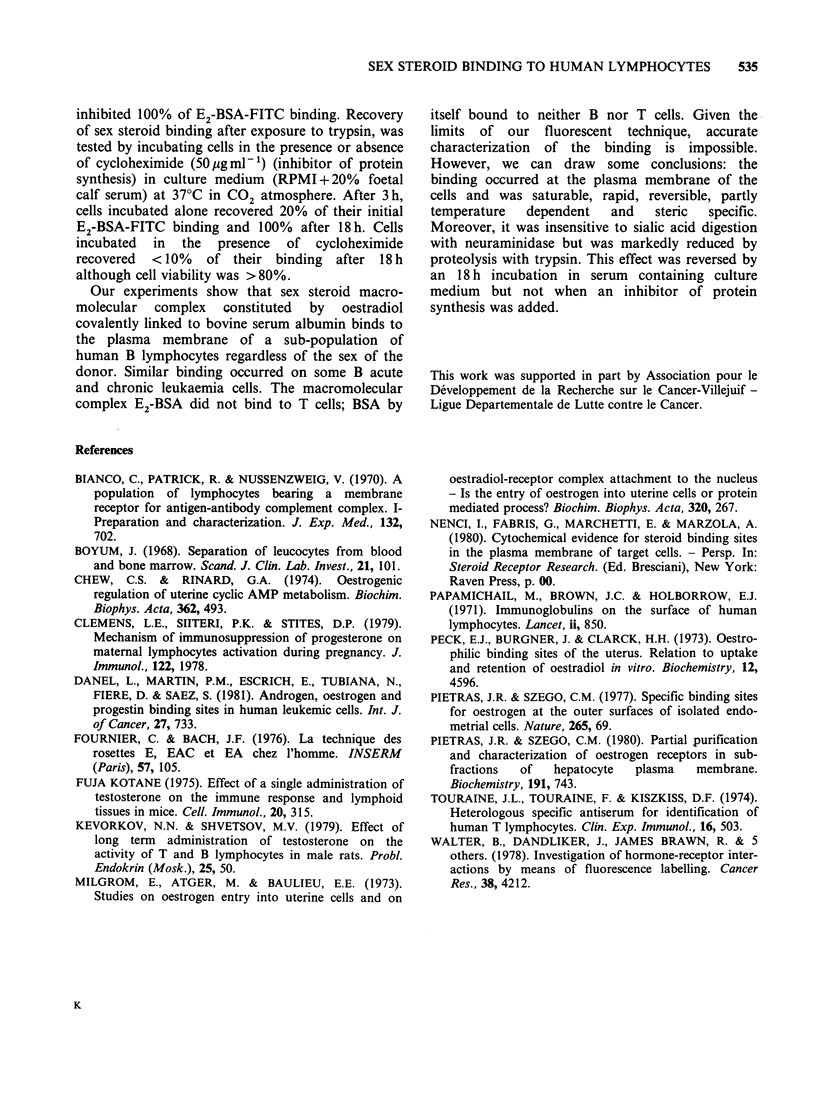# Sex steroid binding to human lymphocytes plasma membrane.

**DOI:** 10.1038/bjc.1984.81

**Published:** 1984-04

**Authors:** N. Tubiana, M. Derre, Y. Carcassonne, P. M. Martin

## Abstract

**Images:**


*Br. J. Cancer* (1984), **49**, 531–536

### Short Communication

### Sex steroid binding to human lymphocytes plasma membrane

N. Tubiana[1], M. Derre[1], Y. Carcassonne[1] & P.M. Martin[2]

[1]*Institut J. Paoli-I. Calmettes,* [2]*Laboratoire de Récepteurs Hormonaux – Faculté Nord, Marseille, France.*

Sex steroid hormones such as testosterone and oestradiol induce immunomodulation of the lymphoid system *in vivo* and *in vitro*, (Clemens *et al.,* 1979; Fuja Kotane, 1975; Kevorkov & Shvetsov, 1979) and cytosolic binding has been demonstrated in normal and pathological human lymphocytes, (Danel *et al.,* 1981). How the hormones enter the cells is controversial: a "passive" diffusion as described by Peck *et al.* (1973) or a "facilitated" diffusion as postulated by Milgrom *et al.* (1973).

Specific binding sites for steroid have been demonstrated on the plasma membrane of target cells (Nenci *et al.,* 1980; Pietras & Szego, 1977, 1980) and steroid binding on their membrane "receptors" induced cellular functions such as cAMP uptake (Chew *et al.,* 1974).

In the present study, human peripheral lymphocytes were characterized with regard to oestradiol membrane binding sites. In order to eliminate diffusion through the membrane and to study external binding, steroid was covalently bound to bovine serum albumin (BSA) to constitute a macromolecular complex which could not diffuse into the cells and this complex was fluoresceinated.

Human peripheral blood from 30 healthy adult donors and from 23 adult patients with chronic or acute leukaemia from the Haematological Department of the Institut J. Paoli-I. Calmettes (Marseille) was collected in heparin. Peripheral blood mononuclear cells (PBMC) were isolated by Ficoll-Hypaque density gradient centrifugation, (Boyum, 1968). From healthy donors, lymphocyte subpopulations were isolated by first removing phagocytic cells with carbonyl iron and second by a Ficoll-Hypaque gradient on rosetted (R) lymphocytes with sheep red blood cells (SRBC) (Fournier & Bach, 1976). The two isolated cells subpopulations R+ cells (T lymphocytes) and R− cells (mostly B lymphocytes) were characterized by SRBC rosettes, surface membrane immunoglobulin (SIg) (Papamichail *et al.,* 1971) and morphological identification by light microscopy after May Grumwald Giemsa Staining.

These characterization techniques yielded the following results: R− cells contained >90% lymphocytes, <5% monocytes and <4% polynuclear cells; 80–90% of these cells were $SIg^+$ and <5% re-rosetted with SRBC.

R+ cells contained >95% lymphocytes <2% monocytes and <2% polynuclear cells. Less than 5% were $SIg^+$ and >80% re-rosetted with SRBC. R+ cells were tested after dissociation of rosettes by ammonium chloride. Viability was >95% in both groups.

R− cells were also tested after incubation with ammonium chloride to eliminate its role in oestradiol membrane binding.

In patient donors with acute leukaemia, poorly differentiated lymphoma or chronic lymphocytic leukaemia, the cells were isolated from peripheral blood before chemotherapy and characterized by the following tests: R and EAC rosette formation, (Bianco *et al.,* 1970), identification of rosetting cells by spinning and staining with May Grumwald Giemsa, cytotoxic assay with an anti-T lymphocyte rabbit antiserum (Touraine *et al.,* 1974) and determination of SIg.

*For oestrogen binding assays* we used oestradiol covalently linked to BSA (steraloids). Each BSA molecule carried on the average 22–25 oestradiol molecules. 1, 3, 5 (10) Estratrien 3,17$\beta$ diol 6 one 6 CMO-BSA was fluoresceinated, ($E_2$-BSA-FITC) (Walter *et al.,* 1978).

Cells ($10^6$) pre-incubated with latex particles in 100 $\mu$l of Hank's medium, were incubated for 30 min at 4°C or 37°C with $E_2$-BSA-FITC at different concentrations, ranging from 0.1 to $4 \times 10^{-5}$ M for the conjugate. In some experiments $E_2$-BSA-FITC was incubated in the presence of dihydrotestosterone (DHT) ($10^{-5}$ M) in the medium. At the end of incubation tubes were centrifuged 10 min at 600 *g* at 4°C and the pellet was washed twice in Hanks solution. After the last wash, the cells were placed on slides, dried in air, fixed with 1% glutaraldehyde, or ethanol; the slides were then mounted in glycerol and examined under an Orthoplan Leitz microscope. The percentage of fluorescent cells was obtained by counting 200 latex

Correspondence: N. Tubiana, Institut J. Paoli-I, Calmettes 232, Boulevard de Sainte Marguerite, 13009, Marseille, France.

Received 19 December 1983; accepted 12 January 1984.

© The Macmillan Press Ltd., 1984

negative cells from each preparation. Controls were incubated with BSA-FITC. Viability of cells was checked at the end of each assay by trypan blue exclusion and was invariably >90%. Each test was run in triplicate and most experiments repeated at least 5 times.

Carbonyl iron-treated peripheral blood mononuclear cells (PBMC) from 30 normal male and female subjects were incubated for 30 min at 4°C or 37°C with $E_2$-BSA-FITC at a concentration of $4 \times 10^{-5}$ M: the average percentage of fluorescent cells was 5.2% (range 0–16%). Fluorescence was bright and distributed over the cell surface. The same test performed on the isolated R+ and R− lymphocyte sub-populations gave significantly different results. In 25 different R− cell populations, the average percentage was 20% (range 8–32), 18% (2–20) in males and 22% (9–32) in females. In contrast, no binding occurred in R+ cell populations. The controls performed with BSA-FITC alone were always negative. In the presence of DHT, similar results were obtained thereby ruling out the binding of $E_2$-BSA-FITC to plasma protein absorbed on the cell membrane. In order to identify the $E_2$-BSA-FITC fluorescent cell sub-population, the same cells were incubated with $E_2$-BSA-FITC and rhodamine goat antibody (Fab′2) to human Ig. (Biolyon). The incubation was performed at 4°C for 30 min to avoid capping of Ig. Interestingly, the cells which were fluorescent for FITC also fluoresced for rhodamine showing that $E_2$-BSA-FITC binding cells were B cells, but when cells were first incubated at 37°C for 180 min with rhodamine Fab′2 antibodies to human Ig and secondly with $E_2$-BSA-FITC, we observed rhodamine capping and diffusely distributed fluorescein on the same cells (Figure 1) $E_2$-BSA-FITC did not interfere with Ig capping mechanisms.

$E_2$-BSA-FITC binding was tested in 23 monoclonal cell populations of chronic and acute lymphocyte leukaemia (Table I). No binding was observed in T or "null" leukaemia. Significant binding occurred only in some B proliferations. In two cases of B acute lymphocytic leukaemia, 70% and 100% of the cells bound $E_2$-BSA-FITC. Consequently normal R− cells or leukaemia B cells were used to characterize the steroid binding. The percentage of $E_2$-BSA-FITC binding cells from the same donor did not change when blood was collected at different times. Furthermore, the freezing time which ranged from one month to one year did not affect binding since it varied only from 27–34% in a 15 test control experiment.

*Saturation binding curve* Cells ($10^6$) were incubated with increasing concentrations of $E_2$-BSA-FITC from 0.1 to $4 \times 10^{-5}$ M. Typical results are shown in Figure 2 where the percentage of fluorescent cells was enhanced from 6% at $0.1 \times 10^{-5}$ M to 20% at

**Figure 1** Rhodamine-FITC membrane staining on the same cell. Photo-micrography of human lymphocytes showing rhodamine fluorescence in a cap and diffuse plasma membrane FITC fluorescence. This double staining was obtained by a double incubation as follows. First R-lymphocytes were incubated with rhodamine-conjugated fragments (Fab′2) antibodies to human Ig for 180 min at 37°C to obtain a rhodamine cap and second, with $E_2$-BSA-FITC $10^{-5}$ M for 30 min at 37°C. After two washes viability was checked by trypan blue exclusion and exceeded 90%. The cells were examined with an Orthoplan Leitz microscope using Block $I_2$ (Leitz) for FITC and Block $N_2$ for rhodamine. The photography was obtained by a superimpression of the both photographs of the same cells.

**Table I** Sex steroid binding to pathological cell populations

| Disease | Patients Identification | Sex | Age | Immunological characterization % of pathol. cells | Lymphocyte subset | From total cell population % $E_2$-BSA fluorescent cells |
|---|---|---|---|---|---|---|
| CLL | RIC | M | 60 | 60 | B ($\gamma\kappa$) | 9 |
| | EYM | F | 50 | 100 | B ($\mu\kappa$) | 74 |
| | CUC | M | 60 | 75 | B ($\mu\delta\kappa$) | 3 |
| | HOL | M | 65 | 100 | B ($\mu\kappa$) | 27 |
| | JAU | M | 42 | 100 | B ($\mu\kappa$) | 29 |
| | LIM | F | 50 | 100 | B ($\mu\kappa$) | 32 |
| | ZER | F | 65 | 60 | B ($\kappa$) | 0 |
| | DER | F | 70 | 80 | B ($\mu\kappa$) | 11 |
| | DIL | M | 50 | 100 | B ($\delta\lambda$) | 36 |
| | BER | M | 60 | 50 | B ($\gamma\kappa$) | 32 |
| | PAC | F | 50 | 80 | B ($\gamma\kappa$) | 11 |
| ALL | DEH | F | 27 | 100 | T | 0 |
| | BES | M | 34 | 100 | T | 0 |
| | LAL | F | 17 | 86 | Not T nor B | 2 |
| | BRE | M | 16 | 100 | Not T nor B | 3 |
| | CAR | F | 72 | 67 | Not T nor B | 5 |
| | DEB | M | 64 | 100 | Not T nor B | 0 |
| | HAN | M | 17 | 96 | Not T nor B | 0 |
| | HEL | M | 14 | 70 | Not T nor B | 0 |
| | LOU | F | 16 | 90 | Not T nor B | 0 |
| PDL | LAT | M | 15 | 80 | B ($\mu\kappa$) | 4 |
| | EST | M | 48 | 95 | B ($\mu\gamma\kappa$) | 9 |

Peripheral blood cells from patients with CLL (chronic lymphocytic leukaemia), ALL (acute lymphocytic leukaemia) or PDL (poorly differentiated lymphoma) were isolated as described in the text.

They were tested by usual immunological methods and identified as:

- B lymphocytes by determination of SIg,
- T lymphocytes by R rosette and/or using an heterologous rabbit antiserum,
- neither T nor B lymphocytes when these tests were negative.

**Figure 2** Saturation binding curve. Percent of fluorescent cells obtained when $10^6$ human normal R-lymphocytes (isolated as described in **Figure 1**) were incubated in Hanks medium with $E_2$-BSA-FITC at concentrations ranging from 0.1 to $4\times10^{-5}$M for 30 min at 37°C. Each $E_2$-BSA-FITC concentration was run in triplicate, the mean values of which are represented by the points shown.

$1\times10^{-5}$M, and remained stable until $4\times10^{-5}$M. Similar binding curves were obtained with several cell populations.

*Determination of binding dissociation* Cell preparations were first incubated with a saturation concentration of $E_2$-BSA-FITC ($4\times10^{-5}$M) for 30 min at 37°C. At the end of incubation, the number of fluorescent cells was counted and regarded as equivalent to 100%. After two washes the second step was performed by incubating these cell preparations with non-fluorescent $E_2$-BSA at different concentrations (from $4\times10^{-6}$M to $1.5\times10^{-4}$M) for 30 min at 37°C. A decrease in the percentage of fluorescent cells was obtained as a function of the concentration of non-fluorescent $E_2$-BSA added (Figure 3).

*Specificity of $E_2$-BSA-FITC binding* Coincubation of $E_2$-BSA-FITC at saturation concentration with non-fluorescent competitors at a 100-fold excess concentration was not possible because these

**Figure 3** Dissociation binding curve. Percentage of fluorescent cells obtained after two incubations: *First:* R-lymphocytes suspended in Hanks medium were incubated with $E_2$-BSA-FITC at $10^{-5}$M (saturation concentration) for 30 min at 37°C and washed twice. The percentage of fluorescent cells determined used as 100%. *Second:* After the two washes, the cells were incubated with non-fluorescent $E_2$-BSA at different final concentrations from $4 \times 10^{-6}$M to $1.5 \times 10^{-4}$M. Each incubation was performed for 30 min at 37°C. At the end of each experiment the percentage of fluorescent cells was determined. *Controls:* the cells were exposed to Hanks medium alone, during the second incubation. Each point represents mean of triplicate determinations.

complexes dissolved poorly in our buffer at these concentrations. Therefore cells were incubated for 30 min at 37°C with non-fluorescent competitors steroid-BSA ($1.5 \times 10^{-4}$M), then $E_2$-BSA-FITC was added ($1 \times 10^{-5}$M) and the incubation was continued for a further 30 min at 37°C. The percentage of fluorescent cells from this double incubation was compared with that obtained after a single incubation with $E_2$-BSA-FITC. The non-fluorescent steroid-BSA conjugates used were: 1,3,5, (10) Estratrien 3,17$\beta$ diol 6 CMO-BSA ($E_2$-BSA) – 1,3,5, (10) Estratrien 3 ol-6,17 dione 6-CMO-BSA ($E_1$-BSA)-4 androsten 17$\beta$ ol 3 one 3 CMO-BSA (Testo-BSA)-4 androsten 3,17 dione, 3 CMO-BSA-4 pregnen 3,20 dione 3 CMO-BSA. These molecules are the same as $E_2$-BSA except in C 17 or C 3 and on A cycle.

Under our experimental conditions, 90% of $E_2$-BSA-FITC binding was inhibited (77–100) by $E_2$-BSA. 50% (45–55) by $E_2$-BSA and less than 20% (0–20) by Testo-BSA and 4 androsten 3,17 dione-BSA as well as pregnen 3,20 Dione-BSA. BSA did not prevent $E_2$-BSA-FITC binding.

*Time temperature and pH dependence of binding* As shown in Figure 4 $E_2$-BSA-FITC binding was maximum after 10 min of incubation at 37°C and was stable up to 360 min. When the steroid was used up to and at saturation concentration ($10^{-5}$M) similar binding kinetics and levels were obtained at 4°C and 37°C. However when the concentration of $E_2$-BSA-FITC used was less than saturation concentration, the binding kinetic was temperature dependent as indicated in Figure 4. The maximum binding was the same at both temperatures but delayed at 4°C. The pattern of immunofluorescence over the cell membrane was studied at 4°C and 37°C. A temperature-dependent phenomenon of capping is known to occur with multivalent ligands bound to plasma membrane of freely suspended cells. This process did not occur with the binding of $E_2$-BSA-FITC to the plasma membrane of lymphocytes.

**Figure 4** Time temperature dependence of binding. Percentage of fluorescent cells obtained after incubation of CLL cells with $E_2$-BSA-FITC from 10 to 300 min at 4°C or 37°C. $E_2$-BSA-FITC was used at saturation concentration ($10^{-5}$M) at 4°C and at 37°C and at a concentration of $5 \times 10^{-7}$M at 4°C and at 37°C. Each point represents mean of triplicate determinations.

Changing the buffer pH from 6.2 to 8.2 which is within the range of cell viability did not noticeably affect binding.

*Effect of enzyme digestion* A preliminary attempt to characterize the $E_2$-BSA-FITC binding sites on lymphocytes, was performed by exposing the cells to enzymes that commonly modify cell membrane receptors: $SIg^+$ lymphocytes were incubated 30 min at 37°C in Hank's medium with trypsin (0.25%) or neuraminidase (10 $\mu$g ml$^{-1}$). The variability checked after each incubation was always >90%. Binding with $E_2$-BSA-FITC was checked as previously stated: neuraminidase had no effect whereas trypsin inhibited 100% of $E_2$-BSA-FITC binding. Recovery of sex steroid binding after exposure to trypsin, was tested by incubating cells in the presence or absence of cycloheximide ($50\,\mu g\,ml^{-1}$) (inhibitor of protein synthesis) in culture medium (RPMI + 20% foetal calf serum) at 37°C in $CO_2$ atmosphere. After 3 h, cells incubated alone recovered 20% of their initial $E_2$-BSA-FITC binding and 100% after 18 h. Cells incubated in the presence of cycloheximide recovered $<10\%$ of their binding after 18 h although cell viability was $>80\%$.

Our experiments show that sex steroid macromolecular complex constituted by oestradiol covalently linked to bovine serum albumin binds to the plasma membrane of a sub-population of human B lymphocytes regardless of the sex of the donor. Similar binding occurred on some B acute and chronic leukaemia cells. The macromolecular complex $E_2$-BSA did not bind to T cells; BSA by itself bound to neither B nor T cells. Given the limits of our fluorescent technique, accurate characterization of the binding is impossible. However, we can draw some conclusions: the binding occurred at the plasma membrane of the cells and was saturable, rapid, reversible, partly temperature dependent and steric specific. Moreover, it was insensitive to sialic acid digestion with neuraminidase but was markedly reduced by proteolysis with trypsin. This effect was reversed by an 18 h incubation in serum containing culture medium but not when an inhibitor of protein synthesis was added.

This work was supported in part by Association pour le Développement de la Recherche sur le Cancer-Villejuif – Ligue Departementale de Lutte contre le Cancer.

### References

BIANCO, C., PATRICK, R. & NUSSENZWEIG, V. (1970). A population of lymphocytes bearing a membrane receptor for antigen-antibody complement complex. I-Preparation and characterization. *J. Exp. Med.*, **132**, 702.

BOYUM, J. (1968). Separation of leucocytes from blood and bone marrow. *Scand. J. Clin. Lab. Invest.*, **21**, 101.

CHEW, C.S. & RINARD, G.A. (1974). Oestrogenic regulation of uterine cyclic AMP metabolism. *Biochim. Biophys. Acta*, **362**, 493.

CLEMENS, L.E., SIITERI, P.K. & STITES, D.P. (1979). Mechanism of immunosuppression of progesterone on maternal lymphocytes activation during pregnancy. *J. Immunol.*, **122**, 1978.

DANEL, L., MARTIN, P.M., ESCRICH, E., TUBIANA, N., FIERE, D. & SAEZ, S. (1981). Androgen, oestrogen and progestin binding sites in human leukemic cells. *Int. J. of Cancer*, **27**, 733.

FOURNIER, C. & BACH, J.F. (1976). La technique des rosettes E, EAC et EA chez l'homme. *INSERM* (*Paris*), **57**, 105.

FUJA KOTANE (1975). Effect of a single administration of testosterone on the immune response and lymphoid tissues in mice. *Cell. Immunol.*, **20**, 315.

KEVORKOV, N.N. & SHVETSOV, M.V. (1979). Effect of long term administration of testosterone on the activity of T and B lymphocytes in male rats. *Probl. Endokrin* (*Mosk.*), **25**, 50.

MILGROM, E., ATGER, M. & BAULIEU, E.E. (1973). Studies on oestrogen entry into uterine cells and on oestradiol-receptor complex attachment to the nucleus – Is the entry of oestrogen into uterine cells or protein mediated process? *Biochim. Biophys. Acta*, **320**, 267.

NENCI, I., FABRIS, G., MARCHETTI, E. & MARZOLA, A. (1980). Cytochemical evidence for steroid binding sites in the plasma membrane of target cells. – Persp. In: *Steroid Receptor Research*. (Ed. Bresciani), New York: Raven Press, p. **00**.

PAPAMICHAIL, M., BROWN, J.C. & HOLBORROW, E.J. (1971). Immunoglobulins on the surface of human lymphocytes. *Lancet*, **ii**, 850.

PECK, E.J., BURGNER, J. & CLARCK, H.H. (1973). Oestrophilic binding sites of the uterus. Relation to uptake and retention of oestradiol *in vitro*. *Biochemistry*, **12**, 4596.

PIETRAS, J.R. & SZEGO, C.M. (1977). Specific binding sites for oestrogen at the outer surfaces of isolated endometrial cells. *Nature*, **265**, 69.

PIETRAS, J.R. & SZEGO, C.M. (1980). Partial purification and characterization of oestrogen receptors in subfractions of hepatocyte plasma membrane. *Biochemistry*, **191**, 743.

TOURAINE, J.L., TOURAINE, F. & KISZKISS, D.F. (1974). Heterologous specific antiserum for identification of human T lymphocytes. *Clin. Exp. Immunol.*, **16**, 503.

WALTER, B., DANDLIKER, J., JAMES BRAWN, R. & 5 others. (1978). Investigation of hormone-receptor interactions by means of fluorescence labelling. *Cancer Res.*, **38**, 4212.